# Clinical Applications of Comprehensive Genomic Profiling in Advanced Non-Small-Cell Lung Cancer—A Case Series

**DOI:** 10.3390/curroncol31060239

**Published:** 2024-05-31

**Authors:** Chun-Ming Tsai, Chih-Hung Lin, Yu-Yen Chou, Hsiao-Yu Jen, Suyog Jain

**Affiliations:** 1 Department of Chest Medicine, Cathay General Hospital, Taipei City 112, Taiwan; linchihhungjhsph@gmail.com (C.-H.L.); yuyenster@gmail.com (Y.-Y.C.); 2 Department of Oncology, Taipei Veterans’ General Hospital, Taipei City 112, Taiwan; 3 Department of Medical Affairs, Guardant Health AMEA, Singapore 138543, Singapore

**Keywords:** case series, cfDNA, guardant 360, liquid biopsy, non-small cell lung cancer

## Abstract

Background: Advanced non-small-cell lung cancer (NSCLC) can be treated with novel targeted therapies that are tailored to the genetic characteristics of malignancy. While tissue-based genomic testing is considered the gold standard for the detection of oncogenic driver mutations, several challenges like inadequate tissue availability, the invasiveness of procuring tumors, and prolonged turnaround time of analysis are encountered. Considering these limitations, guidelines have recognized liquid biopsies using circulating cell-free DNA (cfDNA) as a useful tool to complement conventional tissue testing. Even though cfDNA next-generation sequencing (NGS) can have high sensitivity and specificity, optimal patient benefit requires the interpretation of the molecular profiling results in the context of clinical and diagnostic features to achieve the best outcomes. Case Descriptions: In this case series, we present six patients with advanced NSCLC whose plasma or tissue biopsy samples were analyzed with commercially available comprehensive NGS assays that elucidate the role of testing at various time points in the treatment journey. In all six cases, comprehensive genomic profiling (CGP) provided clinically useful information to guide treatment decisions. Conclusion: Adding to the existing real-world evidence, this case series reinforces that CGP-driven treatment strategies in advanced NSCLC, coupled with other available clinical information, can optimize treatment decisions.

## 1. Introduction

Novel targeted treatments for advanced non-small-cell lung carcinoma (NSCLC) are administered based on the molecular characteristics of the tumor. International treatment guidelines recommend tissue-based genomic testing for potential oncogenic driver mutations involved in NSCLC to guide treatment decisions [1,2,3,4]. In Taiwan, genomic profiling is performed for all newly diagnosed NSCLC patients, and based on the results, targeted therapy is administered. Before starting treatment with immune checkpoint inhibitors (ICI) with or without chemotherapy, the results of PD-L1 tests are considered. One of the challenges in diagnosing advanced NSCLC is the differential landscape of mutational alterations in patients, especially in the Asia Pacific populations versus the Western populations. The proportion of epidermal growth factor receptor (EGFR) mutations is much higher in patients with non-squamous NSCLC in Asia (40–55%) than in the US and Europe (10–15%), while Kirsten rat sarcoma viral oncogene homolog (KRAS) mutations are less common in Asian patients (8–10%) than in Western patients (20–30%) [5,6]. This is further complicated by intratumor and intertumoral heterogeneity and the growing number of genomic biomarkers that should be assessed. 

Delays in diagnosis and treatment further impact NSCLC management, especially in resource-limited settings. Challenges with the use of tissue biopsy for comprehensive genomic profiling (CGP) testing include lack of sufficient tissue for genomic profiling, invasiveness of tumor acquisition, and the time needed to schedule procedures and analyze samples. Furthermore, a single biopsy specimen may not always represent a tumor’s global mutational profile due to intratumor and inter-tumor heterogeneity [7,8]. Considering these shortcomings, guidelines have recognized liquid biopsies using plasma cell-free DNA (cfDNA) in cases where tumor tissue is limited or unavailable, particularly when invasive tissue sampling poses an unacceptable risk or burden [1,2,9,10]. 

The discovery rates for actionable alterations with plasma-based genotyping are similar to tissue-based genotyping [8]. However, since plasma-based genotyping is easily accessible and can be easily applied beyond initial treatment decisions, it can be used to monitor or predict response to therapy [11] and detect mechanisms of tumor resistance at disease progression. 

Here, we present non-consecutive cases of six patients with advanced NSCLC who were monitored prospectively. The plasma or tissue biopsy samples were analyzed with commercially available comprehensive NGS assays as part of standard clinical practice in a single center. All the patients were treated in an academic tertiary care oncology center. 

The objective is to describe the insights that such assays can provide throughout the treatment journey of patients with advanced NSCLC, emphasizing the need to integrate this information comprehensively for appropriate clinical decision-making. 

## 2. Case Presentation

### 2.1. Case 1: Molecular Profiling with Liquid Biopsy Detects Rare Actionable Mutations before Treatment Initiation 

**Figure 1:**
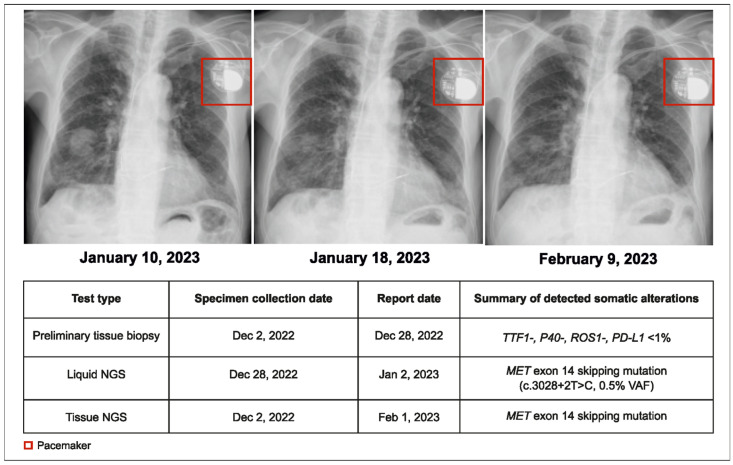
Case 1 details—molecular profiling using liquid biopsy helped in early treatment initiation.

A 95-year-old male patient with NSCLC and pleural effusion in the right lung but with no distant metastases (cT3N3M1a, stage IVA) was presented to the physician. The preliminary pathology report from tumor tissue testing indicated no actionable alterations (wild type for EGFR, ALK, and ROS1). Following this, both tissue and plasma CGP were conducted. A MET exon 14 (METex14) skipping mutation was detected by the plasma CGP test within 5 days. Treatment with tepotinib 225 mg twice daily (BID) was initiated within 14 days of the initial consultation (Figure 1). 

More than one month after the original request, the tissue NGS results corroborated the plasma CGP findings. Unlike plasma CGP, which was performed in a single center, tissue testing was performed at various laboratories depending on real-world practice, and subsequently, it took longer to receive tissue NGS results than plasma NGS. The tumor showed partial response to therapy for 8 months, and the patient died due to aspiration pneumonia. This case demonstrates the shorter turnaround time (TAT) of plasma CGP and its reliability in detecting rare but actionable genomic alterations, leading to early treatment initiation. 

### 2.2. Case 2: CGP Identifies an Actionable Mutation in a Patient with PD-L1 Positive NSCLC Who Had Rapid Disease Progression during Immune Checkpoint Inhibition 

**Figure 2:**
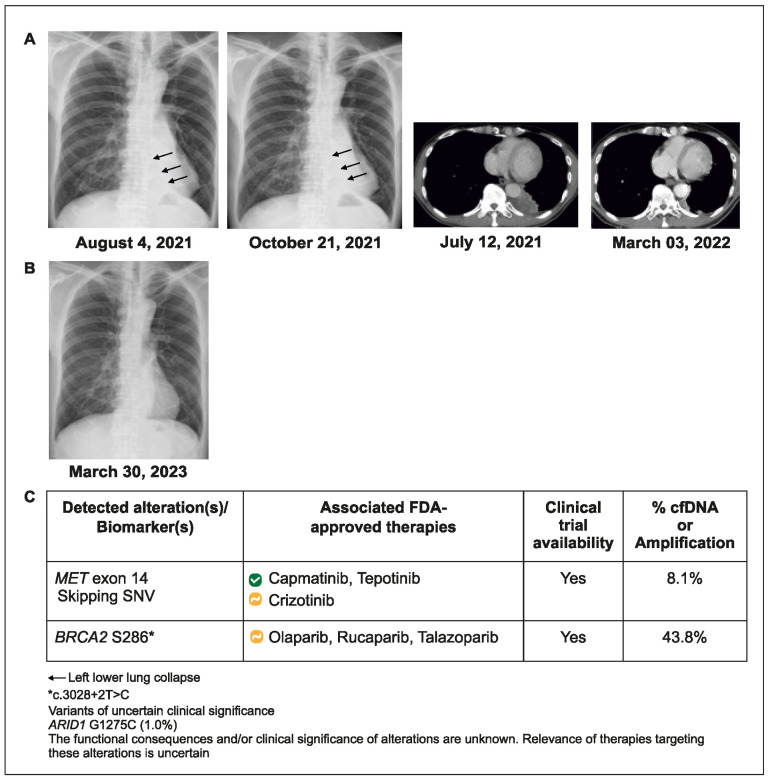
Case 2 details—(**A**) Tumor with airway obstruction and left lower lung collapse (indicated by the arrows); (**B**) After treatment, tumor regression with re-expansion of left lower lung; (**C**) Molecular profiling helped in better understanding of patient’s genetic code.

A 70-year-old man with advanced NSCLC and metastases to the lung and brain underwent standard tissue hotspot testing for EGFR, ALK, and ROS 1, with no actionable alteration detected in EGFR, ALK, or ROS1. No NGS was performed on the initial tissue biopsy. Since tumor PD-L1 staining was >95%, he was treated with pembrolizumab plus bevacizumab. After 1.5 months of treatment, disease progression occurred, accompanied by left lower lung collapse. Blood-based CGP testing was ordered, and METex14 skipping mutation was detected within 7 days. The patient’s treatment was switched to capmatinib for 5 months (showing partial response), followed by tepotinib (due to declining renal function), which remains ongoing after 1 year (Figure 2). 

This case reveals that relying on tissue tests targeting hotspot mutations might overlook actionable alterations while retesting for genomic biomarkers during rapid disease progression could detect previously unidentified actionable alterations.

### 2.3. Case 3: CGP Detects Potential Primary Resistance Mutations to Immunotherapy

**Figure 3:**
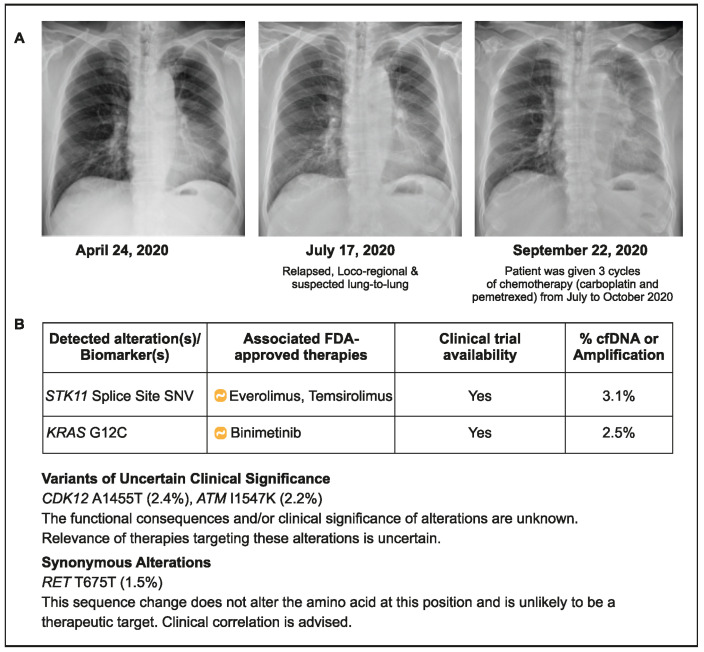
Case 3 details—(**A**): Showing chest X-rays at various time points; (**B**) Results of liquid CGP testing conducted in October 2020.

A 70-year-old male patient (ex-smoker) was initially diagnosed with stage IIB NSCLC without any actionable alteration in EGFR or ALK (both wild type). The patient underwent surgical resection of the tumor in September 2019, followed by four cycles of adjuvant chemotherapy (vinorelbine/cisplatin). He presented again with cough and dyspnea and was diagnosed with relapsed advanced NSCLC with a suspected lung metastasis in July 2020. Based on the PD-L1 status (5%) of the archived tumor sample, he was treated with pembrolizumab plus chemotherapy (carboplatin and pemetrexed) for 3 months (July 2020–October 2020). Due to apparent progression, plasma CGP testing was conducted in October 2020, and a serine/threonine kinase 11 (STK11) splice site mutation with KRAS G12C was detected (Figure 3). The presence of STK11 and KRAS co-mutations may have contributed to the lack of improvement seen with ICI use in this patient. This reinforces the need for CGP before treatment initiation to identify patients who may be less likely to benefit from immunotherapy. However, in practical terms, the choice of medicines would not have changed in the current situation due to the lack of approved drugs targeting STK11 KRAG12C first line and the genomic profiling information for the next line of treatments.

### 2.4. Case 4: Serial cfDNA Testing Detects Emerging Actionable Resistance Alterations 

**Figure 4:**
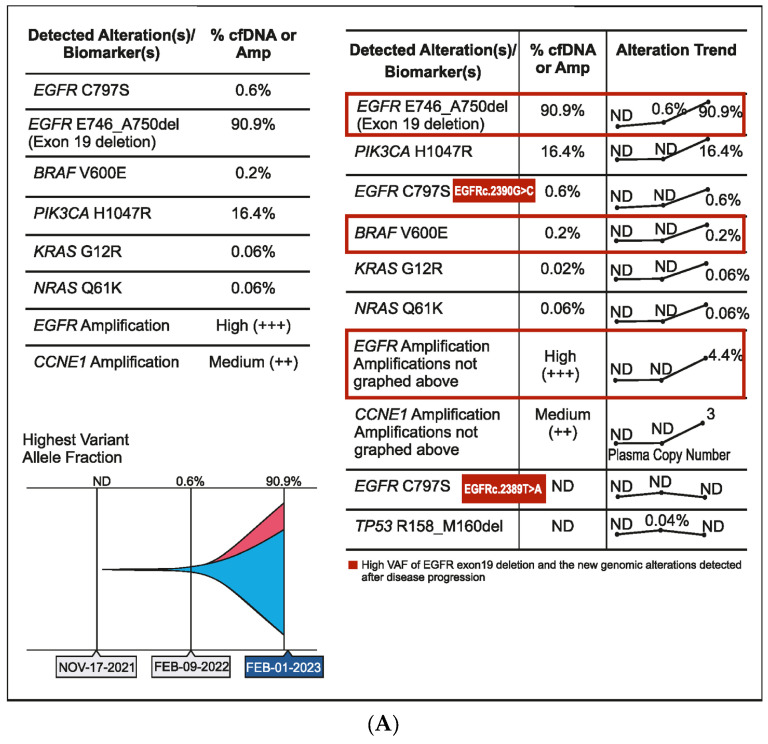

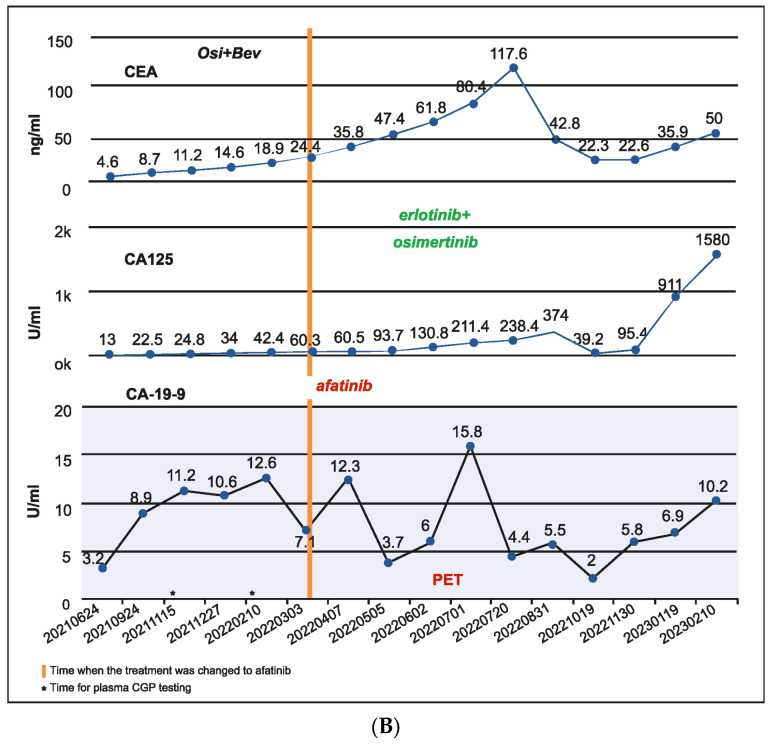
(**A**) Case 4 details—Molecular profiling at different time points demonstrating the clonal dynamics of multiple tumor subclones. (**B**) Case 4 details—Serum biomarker levels at different time points.

A 56-year-old male patient was diagnosed in September 2020 with EGFR-mutated (exon 19 deletion) advanced NSCLC. He had lung, brain, and bone metastases along with pleural effusion and was treated with osimertinib. Drainage of the pleural effusion was performed three months after the initial diagnosis, and bevacizumab was added to osimertinib. An increase in serum carcinoembryonic antigen (CEA) (11.2 ng/mL), cancer antigen 19-9 (CA19-9) (11.2 U/mL), and cancer antigen 125 (CA 125) (24.8 U/mL) was detected after a year of treatment and no changes observed on the scan. However, plasma CGP testing showed no tumor-related somatic alterations. Three months later, as CEA (18.9 ng/mL) and CA125 (42.4 U/mL) levels continued to rise, but no changes were observed on the scans, a second plasma CGP test was conducted. An EGFR exon 19 mutation (E746_A750 deletion; 0.6% VAF) was detected along with two subclones encoding EGFR C797S (EGFRc.2390G > C, variant allele frequency (VAF) 0.1%; EGFRc.2389T > A, VAF 0.04%), which were confirmed to be in trans to the driver mutation (Figure 4A). Treatment was changed to afatinib in March 2022, followed by osimertinib plus erlotinib in June 2022 for two months. Switching of treatment to osimertinib plus erlotinib was carried out because there was no improvement with osimertinib plus bevacizumab and EGFR C797S mutation (resistant to third-generation EGFR TKIs but sensitive to first- and second-generation EGFR TKIs) was detected in the patient. However, CEA (61.8 ng/mL) and CA125 (130.8 U/mL) levels continued to increase. The patient showed no improvement in symptoms and had abdominal pain and diarrhea. In July 2022, a PET scan detected peritoneal metastasis, and chemotherapy (pemetrexed/carboplatin/bevacizumab) was administered. Six months later, serum CA125 increased to 1580 ng/mL, and a third liquid CGP testing was ordered. EGFR amplification was detected along with EGFR exon 19 deletion and one of the previously detected EGFR C797S subclones (EGFRc.2390G > C) along with other new genomic alterations, including PIK3CA H1047R (VAF 16.4%), BRAF V600E (VAF 0.2%), KRAS G12R (VAF 0.06%), NRAS Q61K (VAF 0.06%), and CCNE1 amplification (medium plasma copy number 3) (Figure 4A,B). Accordingly, the treatment regimen was switched to a combination of afatinib (30 mg), cetuximab (150 mg/m^2^ every two weeks), dabrafenib (75 mg BID), and trametinib (2 mg QD) due to the detection of BRAF V600E and EGFR amplification after disease progression. The patient showed a dramatic response in one week but succumbed to a myocardial infarction after one month of therapy.

### 2.5. Case 5: Detection of Actionable Alteration in a Tissue Biopsy after Disease Progression and Persistently Negative Liquid Biopsy Results

**Figure 5:**
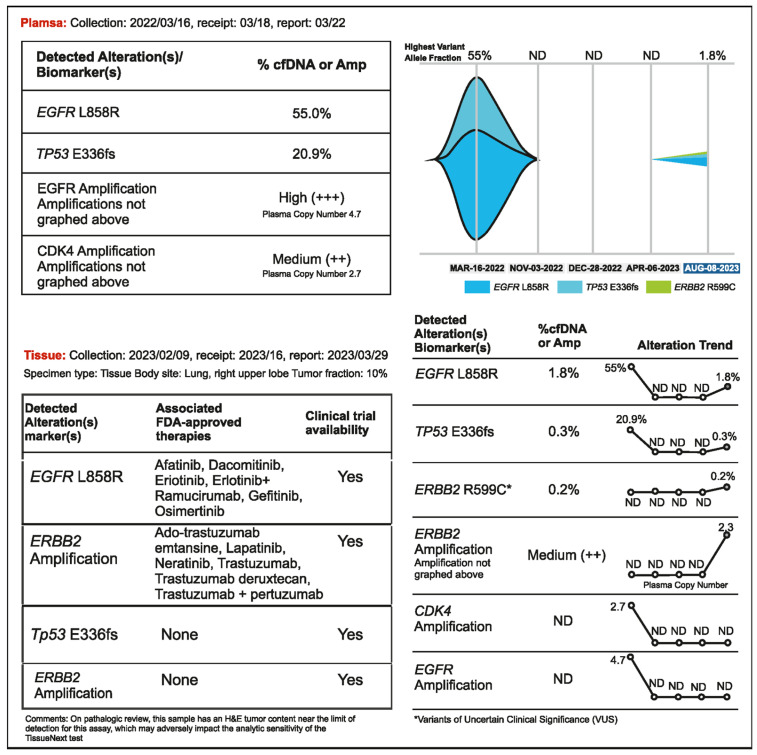
Case 5 details—Importance of tissue biopsy-driven molecular profiling in clinical decision.

A 56-year-old woman presented with advanced NSCLC manifested as multiple bone and brain metastases. Liquid biopsy detected EGFR L858R (VAF 55%) and the patient was treated with osimertinib (80 mg daily), which resulted in stable disease for about eight months. A routine brain scan in November 2022 (performed every 3 months) showed no improvement; hence, bevacizumab was added to the regimen. A plasma CGP test did not detect the presence of tumor DNA, but the CEA level increased to 47.9 ng/mL. After one month of bevacizumab administration, no change was observed in brain and lung lesions; hence, the osimertinib dose increased to 160 mg in January 2023. Following this, a regular PET scan showed an active but stable lesion in the lung. CEA remained elevated (64 ng/mL). The lung lesion was resected in February 2023, but there was a persistent increase in CEA levels. This prompted the treating physician to order tissue NGS testing from the resected right upper lobe lesion and another liquid biopsy in April 2023. No tumor DNA was detected in plasma; however, EGFR L858R and ERBB2 amplification (copy number 21.36) were found in tumor tissue (Figure 5). Osimertinib and bevacizumab were administered, and the patient remained stable until bone and lymph node relapse was detected in a routine PET scan in August 2023. ERBB2 amplification (plasma copy number 2.3) was detected by liquid biopsy at that time. In this case, even though the tumor was metabolically active on a PET scan, detectable levels of tumor DNA were not being shed into the bloodstream. Surprisingly, no cfDNA was detected, not even the driver mutation, when the original VAF on cfDNA NGS was very high (55%). Osimertinib may have kept the previously shedding tumors in check but had no impact on the clones with ERBB2 amplification, which were not shedding DNA. In such cases, analysis of a tumor tissue sample may be needed to detect new and potentially actionable genomic alterations.

### 2.6. Case 6: Serial Monitoring Detects Actionable Molecular Profiles throughout the Disease

**Figure 6:**
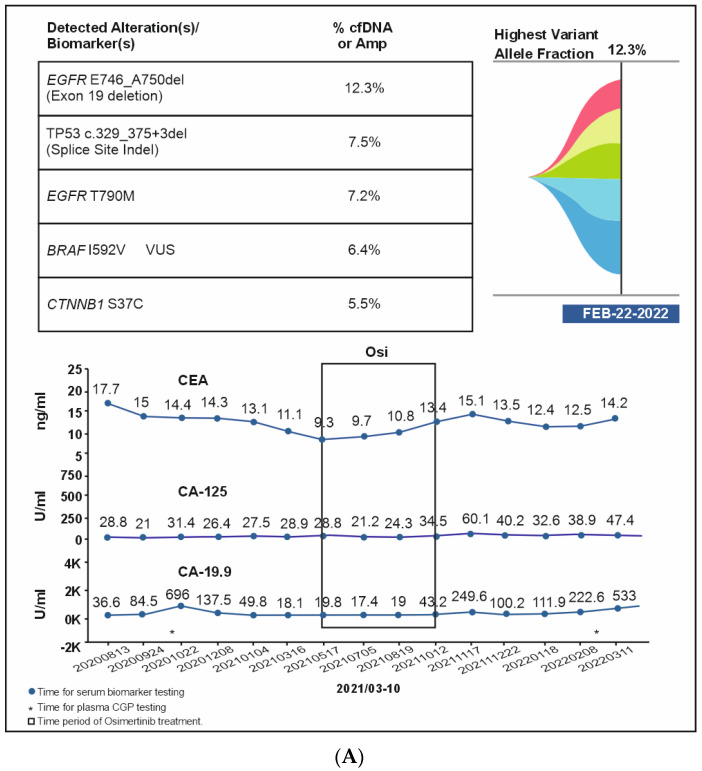

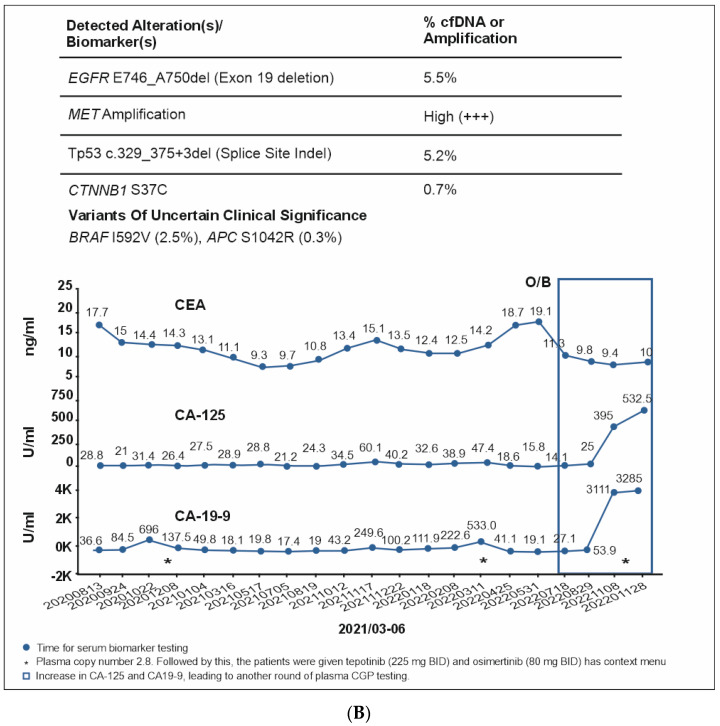

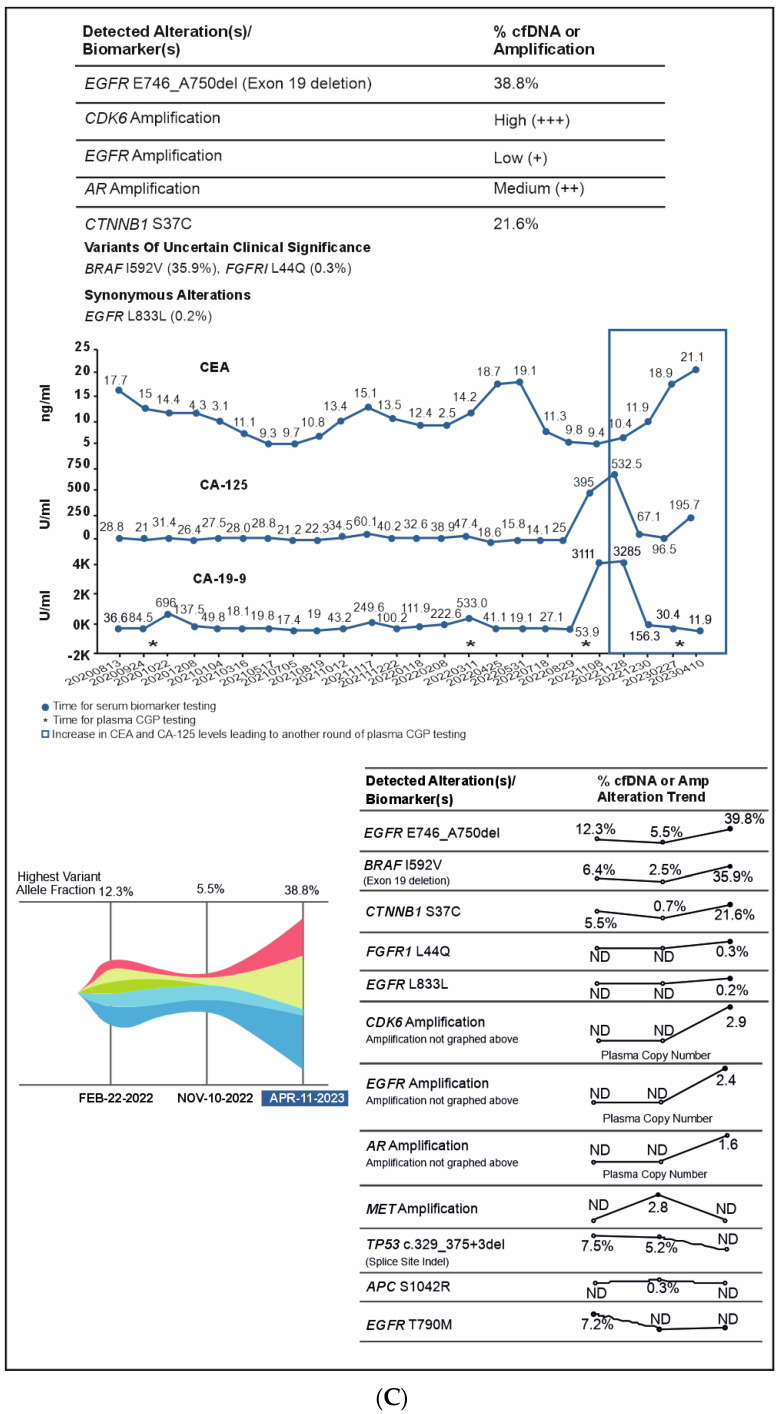

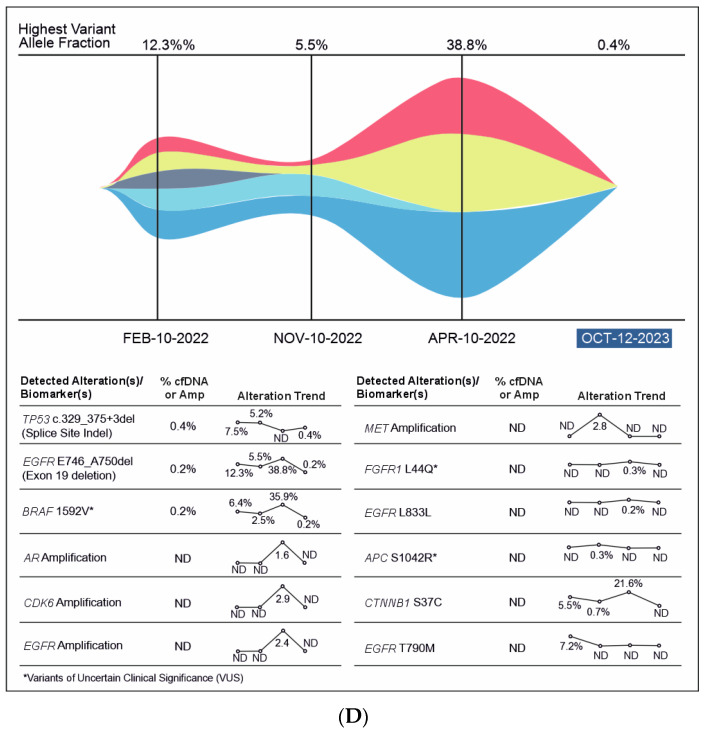

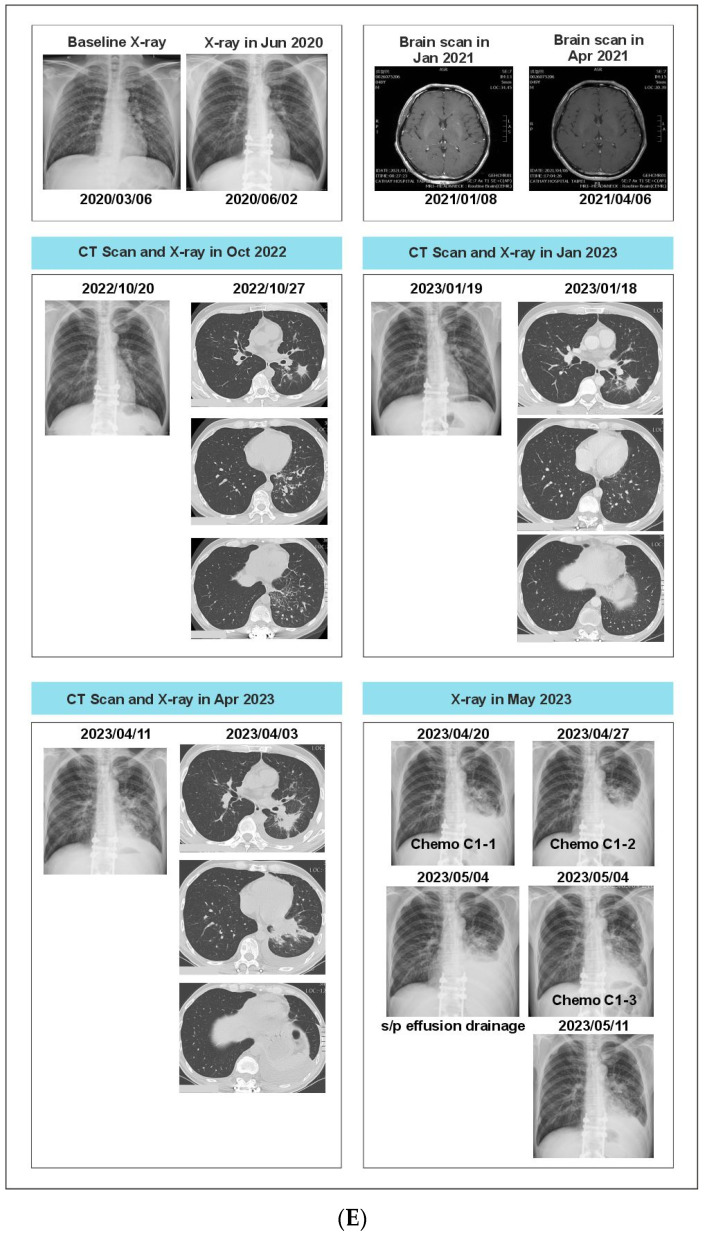
(**A**): Case 6 details—Serum biomarker levels and tumor mutational spectrum at first CGP testing. (**B**): Case 6 details—Serum biomarker levels and tumor mutational spectrum at second CGP testing. (**C**): Case 6 details—Serum biomarker levels and tumor mutational spectrum at third CGP testing. (**D**): Case 6 details—Serum biomarker levels and tumor mutational spectrum at fourth CGP testing. (**E**): Case 6 details—Imaging results at different time points.

A 51-year-old non-smoking male patient was diagnosed with EGFR-mutated (exon 19 deletion) advanced NSCLC with metastases to the lung, brain, and bone. Baseline serum CA19-9 and CA125 were within normal limits. He was treated with afatinib for three months, followed by afatinib plus bevacizumab for the next six months (Supplementary Figure S1). In Oct 2020, an increase in CA19-9 was noted (696 U/mL) along with bone pain, and a liquid biopsy was conducted thereafter. Results showed only ERBB2 R143Q. Cetuximab was added to afatinib. After a dramatic reduction in CA19-9 levels (to 137 U/mL) but the development of a new nodule in the brain, the treatment regimen was changed from bevacizumab to afatinib plus bevacizumab to osimertinib. Within three weeks of osimertinib administration, the new brain lesion was no longer detected on magnetic resonance imaging (Figure 6). Osimertinib was administered for 6 months until the serum concentration of three tumor biomarkers reached normal limits (CA19-9: 249 U/mL, CA-125: 60.1 U/mL; CEA: 15.1 ng/mL). The treatment was then switched to pemetrexed plus carboplatin and bevacizumab followed by afatinib plus bevacizumab. A month later, plasma CGP was ordered. Liquid biopsy results at different time points are depicted in Figure 6A–D, and imaging results are shown in Figure 6E. Results revealed the presence of EGFR T790M along with the known EGFR exon 19 deletion (Figure 6). The patient was treated with four cycles of pemetrexed with carboplatin and bevacizumab followed by osimertinib and bevacizumab for four cycles (Figure 6). An increase in CA19-9 and CA-125 after four months was noted, leading to another round of plasma CGP testing; results revealed MET amplification. The patient was shifted to tepotinib and osimertinib for four months. However, there was an increase in CEA; new CGP tests detected high CDK6 and EGFR amplification (Figure 6). The patient was switched to osimertinib and paclitaxel (60 mg) (Supplementary Figure S1). After 6 months, a fourth liquid CGP test detected EGFR mutation (exon 19 deletion). This case demonstrates how dynamic molecular monitoring using cfDNA NGS can assist in treatment decisions. 

## 3. Discussion

Developments in precision oncology have prompted the evaluation of novel diagnostic tools to overcome some of the limitations of traditional tumor genotyping. The minimally invasive liquid biopsy techniques allow real-time biomolecular characterization of the tumor through the analysis of human body fluids [12]. In the current case series, we presented six patient scenarios in which tumor molecular profile was assessed in real time and how CGP results were integrated with other clinical data to aid the management of patients with advanced NSCLC. 

The first patient case demonstrated how liquid biopsy-driven CGP tests were quicker in providing diagnosis (and hence treatment) than tissue biopsy and that plasma-based NGS has comparable diagnostic accuracy to tissue-based genotyping [8]. This is consistent with earlier studies that support the use of liquid biopsy-driven CGP testing in advanced NSCLC for treatment decisions [10,13,14]. 

Testing for common abnormalities in a single gene can overlook less common but actionable driver alterations in other genes [15]. This was exemplified in both the first and second patient case studies, in which liquid CGP detected METex14 skipping (actionable mutation) that was not found by standard tumor tissue analysis. 

One concern in NSCLC treatment is interpreting molecular profiling results, especially in the presence of KRAS mutation. KRAS gene is the most frequently mutated oncogene in human cancers, with KRAS G12C occurring in 13% of all NSCLC cases in Western populations [16,17]. However, among Taiwanese NSCLC patients, a much lower prevalence of KRAS mutation is observed, with only 7.7% having any KRAS mutation and 2.5% of all cases with KRAS G12C [18]. Furthermore, mutations in tumor suppressor genes TP53 and STK11 are common in lung adenocarcinoma and frequently co-occur with KRAS mutations [19,20]. An earlier study demonstrated that patients with TP53 and/or KRAS mutation showed sensitivity to PD-L1 blockade only when there was no loss of function mutation of STK11 and KEAP1 [20]. Although the first-line treatment of patients with KRAS mutations in NSCLC is still ICI or platinum-based chemotherapy, based on clinical trial data, KRAS-targeted drug sotorasib or adagrasib has been approved by US Food and Drug Administration for the treatment of patients with KRAS G12C mutations who had received at least one previous systemic therapy (chemotherapy or ICI or both) and had disease progression [21,22,23]. It has been reported that STK11/KRAS co-mutated patients were not associated with improved survival following ICI therapy, and KRAS/STK11 co-mutations may predict primary resistance to ICI [24]. Consistent with these findings, the third patient case scenario reflects the importance of obtaining a holistic overview of the tumor molecular profile before selecting treatment. This case indicated how CGP results can be used to identify patients who may not be responsive to immunotherapy. 

In response to treatment, the molecular profile of NSCLC can be dynamic. This is reflected in the fourth case. The tumor of this patient developed treatment resistance that could be monitored using serum biomarkers at different time points, and utilizing CGP at relevant time points in the treatment journey helped in clinical decisions. This case also demonstrates the molecular heterogeneity between primary lesions and their corresponding metastases, which can lead to mixed tumor responses to targeted treatment [25].

Although both clinicians and patients prefer liquid biopsy over tissue biopsy [26], it is not a complete replacement for tumor tissue testing, as demonstrated in the fifth patient case, where plasma CGP failed to identify an actionable mutation. Similar observations have confirmed that cfDNA NGS and tissue NGS are not perfect alone, and each could miss alterations [27]. Although liquid biopsies are preferred diagnostic options at the time of disease progression, this case supports the recommendation of most guidelines that tissue biopsy should be conducted when liquid biopsy results are negative [28].

The main goal in the clinical management of advanced NSCLC is to control tumor progression while maintaining an acceptable quality of life for the individual patient. Serum tumor biomarkers (such as CEA, CA19-9, CA125), released by tumor cells or immune cells in response to tumor growth, play an important function in clinical diagnosis, prognosis, and anti-drug surveillance, as well as in predicting therapeutic outcomes in NSCLC. They are found to be useful in measuring chemotherapy response [29]. Additionally, these biomarkers can be employed as useful prognostic indicators in addition to PD-L1 in ensuing clinical applications. Moreover, their routine measurement in clinical settings may serve as a simple and reliable method for estimating the effectiveness of immunotherapy [30]. Furthermore, imaging tools are routinely used for diagnosis, staging, and monitoring the clinical response to an intervention in lung cancer management. Although these diagnostic tools alone may not be useful in treatment decisions due to their low sensitivity and specificity, they can help in appropriate interventional decisions when used in combination with CGP. Many studies of cfDNA NGS focus on biomarker discovery rates, but some correlate biomarker detection to the clinical efficacy of targeted therapies [31,32,33,34]. In the sixth patient scenario, elevations in serum biomarker levels were correlated with changes in actionable mutations and guided treatment decisions throughout the patient’s disease course. It is thus essential to consider all available diagnostic options, including CGP of tissue and blood, imaging, and serum biomarkers, to obtain a complete picture of the patient’s disease and to guide clinical judgment.

The current study discussed the features of individual patient cases. The sample size is small, and the descriptions are anecdotal. Nevertheless, these real scenarios represent typical cases that may be found in clinical practice and provide examples of how CGP testing can be incorporated into standard practice for patients with advanced NSCLC. In countries where drugs are approved based on trials and guidelines, and their use/reimbursement is strictly restricted to approved indications and lines of therapy, the treatment sequences received by patients 4, 5, and 6 would not have been possible.

## 4. Conclusions

In conclusion, this case series on six patients supports the incorporation of CGP-driven treatment strategies in advanced NSCLC irrespective of the source of biopsy, although confirmation is warranted in additional patients. The findings also suggest that understanding the dynamics of the tumor microenvironment and interpreting the CGP results in consideration with other available diagnostic tools like serum biomarkers and imaging can help in better treatment decisions.

## Data Availability

The original contributions presented in the study are included in the article/Supplementary Material, further inquiries can be directed to the corresponding author.

## Supplementary Materials

The following supporting information can be downloaded at: https://www.mdpi.com/article/10.3390/curroncol31060239/s1, Figure S1: Case 6 timeline.
