# Supplementary figures and images for: Clinical Applications of Comprehensive Genomic Profiling in Advanced Non-Small-Cell Lung Cancer—A Case Series
Source: Curr Oncol. 2024 May 31;31(6):3161–76. doi: 10.3390/curroncol31060239 (PMC11202974; doi:10.3390/curroncol31060239)

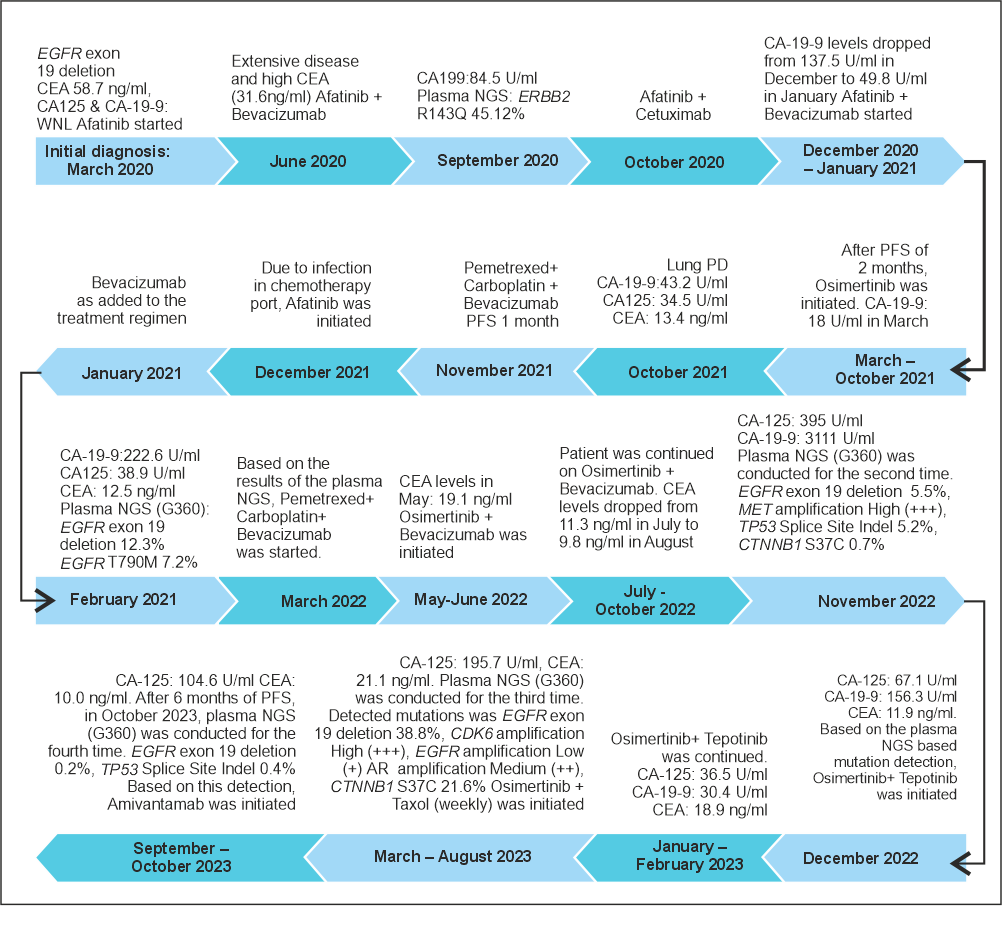

Supplement: Supplementary file 1 [file curroncol-31-00239-s001.zip › curroncol-3007836-supplementary.png]
